# Differences in lipidomics may be potential biomarkers for early diagnosis of pancreatic cancer^1^

**DOI:** 10.1590/s0102-865020200050000008

**Published:** 2020-07-06

**Authors:** Dehua Zhou, Di Mu, Ming Cheng, Yuting Dou, Xianwei Zhang, Zhensheng Feng, Guangting Qiu, Hua Yu, Yang Chen, Hong Xu, Jian Sun, Ling Zhou

**Affiliations:** I Master, Department of General Surgery, Shanghai Fourth People’s Hospital Affiliated to Tongji University School of Medicine, Shanghai, China. Statistical analysis, conception and design of the study, manuscript preparation.; II Master, Department of Clinical Laboratory, Shanghai Fourth People’s Hospital Affiliated to Tongji University School of Medicine, Shanghai, China. Acquisition, analysis and interpretation of data; statistical analysis; conception and design of the study; manuscript preparation.; III Master, Department of General Surgery, Shanghai Fourth People’s Hospital Affiliated to Tongji University School of Medicine, Shanghai, China. Intellectual, scientific, conception and design of the study; acquisition, analysis and interpretation of data; technical procedures; statistical analysis; manuscript preparation, final approval.; IV Bachelor degree, Department of Clinical Laboratory, Shanghai Changning Maternity and Infant Health Hospital, Department of Clinical Laboratory, China. Technical procedures, critical revision.; V Bachelor degree, Department of General Surgery, Shanghai Fourth People’s Hospital Affiliated to Tongji University School of Medicine, Shanghai, China. Technical procedures.; VI Bachelor degree, Department of General Surgery, Shanghai Fourth People’s Hospital Affiliated to Tongji University School of Medicine, Shanghai, China. Acquisition, analysis and interpretation of data.; VII Master degree, Department of Neurosurgery, Shanghai Fourth People’s Hospital Affiliated to Tongji University School of Medicine, Shanghai, China. Acquisition, analysis and interpretation of data.; VIII Department of General Surgery, Shanghai Fourth People’s Hospital Affiliated to Tongji University School of Medicine, Shanghai, China. Intellectual, scientific, conception and design of the study; final approval.; IX MD, Department of General Surgery, Shanghai Fourth People’s Hospital Affiliated to Tongji University School of Medicine, Shanghai, China. Acquisition, analysis and interpretation of data; technical procedures; critical revision; manuscript preparation, final approval.

**Keywords:** Pancreatic Neoplasms, Lipidomics, Diagnosis, Biomarkers

## Abstract

**Purpose:**

To analyze the plasma lipid spectrum between healthy control and patients with pancreatic cancer and to select differentially expressed tumor markers for early diagnosis.

**Methods:**

In total, 20 patents were divided into case group and healthy control group according to surgical pathology. Of almost 1206 plasma lipid molecules harvested from 20 patients were measured by HILIC using the normal phase LC/MS. Heat map presented the relative levels of metabolites and lipids in the healthy control group and patients with pancreatic cancer. The PCA model was constructed to find out the difference in lipid metabolites. The principal components were drawn in a score plot and any clustering tendency could be observed. PLS-DA were performed to distinguish the healthy control group and pancreatic cancer according to the identified lipid profiling datasets. The volcano plot was used to visualize all variables with VIP>1 and presented the important variables with P<0.01 and |FC|>2.

**Results:**

The upregulated lipid metabolites in patients with pancreatic cancer contained 9 lipids; however, the downregulated lipid metabolites contained 79 lipids.

**Conclusion:**

There were lipid metabolomic differences in patients with pancreatic cancer, which could serve as potential tumor markers for pancreatic cancer.

## Introduction

Pancreatic cancer is a deadly disease with a rising incidence, and it will become the second leading cause of tumor-related death in some areas. There were an estimated 55,400 new cases of pancreatic cancer in the United States in 2018, with 44,330 deaths^1^. Pancreatic cancer had a low 5-year survival rate (8%) in solid tumors mainly because of the difficulty in early diagnosis. Radical resection was the first choice for pancreatic cancer; however, most patients had already advanced tumors when they were first diagnosed^2,3^. Only 15-20% of patients with pancreatic cancer were eligible for radical surgical resection, so the core task of improving the surgical resectability of pancreatic cancer was to diagnose it earlier^4^.

Lipidomics was one of the metabolomics approach that focuses on lipids and was a promising technique for overviewing lipid profiles in blood and tissues^5-7^. It has been reported that the denovo synthesis of fatty acids was significantly higher in tumor cells and tumor tissues than that of normal cells to meet the needs of rapid growth and expansion of cells. In addition, cancer cells also accumulated lipids in the form of lipid drop^8^. However, few studies have been reported on lipid metabolism in pancreatic diseases. Lipid spectrum is helpful to understand the pathophysiological mechanism of disease and to explore its specific biomarkers; this was because lipids had a wide variety of biological functions, including protective membrane integrity, energy storage, signal transduction, and involvement in cell growth, proliferation, and death^9-12^.

Mené et al reported that lipogenic enzymes were consistently overexpressed in many cancer types including pancreatic cancer^9^. Lipids could sufficiently promote the proliferation of pancreatic cancer cells lines; meanwhile, tumors could also activate the denovo synthesis of fatty acids regardless of the levels of circulating lipids. More than 93% triacylglycerol fatty acids were obtained by denovo synthesis and were activated by several signaling pathways^7^. Given lipidomics plays a major role in the development, evolution, metabolism and mechanic function of pancreas and pancreatic disease, we present our study on pancreatic cancer and heterogeneity of lipidomic profiles. Thus, we aimed to analyze the plasma lipid spectrum between healthy control and patients with pancreatic cancer and to select differentially expressed tumor markers for early diagnosis.

## Methods

A case-control study that included 20 patients admitted into the Department of General Surgery, Shanghai Fourth People’s Hospital was conducted during November 2018 to December 2018. The protocol of this study was approved by the Institutional Review Board of Shanghai Fourth People’s Hospital Affiliated to Tongji University School of Medicine (No.2019057-001). Written informed consent was obtained from each participant.

The case group was composed of 14 patients with pancreatic cancer who were clinically preliminary diagnosed by imaging examination (CT or MRI) combining with serum tumor marker and then finally confirmed by surgical pathology. The control group was composed of 6 healthy patients with regular physical examination. The mean age was 57.50 year in the control group and 60.21 year in patients with pancreatic cancer. There were 3 patients with T2N0Mx, 4 patients with T2N1Mx, 3 patients with T2N2Mx and 4 patients with T3N1Mx. According to the postoperative pathological results, 10 cases were moderately differentiated ductal adenocarcinoma and 4 cases were poorly differentiated ductal adenocarcinoma.

The exclusion criteria for both patients in the case group and the control group were the diagnosis of chronic pancreatitis, periampullary tumor, cholangitis, hyperlipemia, and prior to taking of statins. Also excluded were patients who performed chemotherapy or blood transfusions within the last 6 months.

### *Sample collection*

Plasma samples were separated from whole blood that was obtained by venipuncture for 14 patients with pancreatic cancer and 6 healthy controls. The plasma samples from patients with pancreatic cancer were collected in the week 1 before interventions. Of the 14 patients, 12 underwent pancreaticoduodenectomy and 2 underwent resection of the body and tail of pancreas plus splenectomy. Plasma for 20 patients was initially stored at −80°C (frozen pipe, containing ethylenediamine tetraacetic Acid).

### *Lipid analysis*

Of almost 1206 plasma lipid molecules harvested from 20 patients were measured by Hydrop Interaction Liquid Chromatography (HILIC) using the normal phase High-performance Liquid Chromatography-Mass Spectrometry (HPLC/MS).

The modified Bligh & Dyer method was used to extract total fat from 200ul plasma. The internal standard cocktail (Avanti Lipids Polar) was added in 10uL to per sample number, and lipid extracts were subjected to the normal-phase silica liquid chromatography-coupled triple-quadrupole mass spectrometers (Qtrap^®^ 4000 and 6500, Sciex, Framingham, MA, USA). Using positive and negative ESI modes, Q-trap operates in MRM mode, scanning for different precursor/product ion pairs. Each experiment was repeated three times. MultiQuant™ software (AB Sciex) processes the MRM data, and the peak area of each pair is used for further quantification. Frozen samples were thawed at room temperature and 0.1mL of each plasma sample was placed in a 2ml Eppendorf vial (Fisher Scientific). To precipitate proteins, methanol (2 mL), water (0.9 mL) and dichloromethane (0.9 mL) were added and the mixture was vortexed for 5 seconds. 0.1ml of the internal standard mixture was added and left at room temperature for 30 minutes. 1ml of water and 0.9 ml of dichloromethane were added. Next, the mixture at 20800 g was centrifuged for 10 minutes and the bottom organic layer was collected into a new Eppendorf bottle. The supernatant was mixed with 1.8ml dichloromethane, vortex for 5 seconds, centrifuged at 20800g for 10 minutes, recovering the remaining liposomes in the original extraction tube, and mixing the bottom layer with the liposomes collected after the first centrifugation. The composite substrates for each sample were concentrated under nitrogen and recombined in 0.25ml of the operating solution (10mM ammonium acetate, dichloromethane (50): methanol (50)). Quality control (QC) is a mixture of serum samples from all two groups of subjects, which were also processed and prepared in the same manner as individual samples, and analyzed using plasma samples for every 10 samples. The samples were transferred to test tubes for LC-MS analysis. Briefly, LC-MS/MS analysis was performed on an Agilent 1260 LC (Agilent Technologies, Santa Clara, CA) AB Sciex QTrap 5500 MS (AB Sciex, Toronto, Canada) system. MRM transfer was monitored in both negative and positive modes using MultiQuant 2.1 software (AB Sciex, Toronto, Canada) to integrate extracted MRM peaks.

### *Statistical analysis*

All of these statistical methods were performed by R sofware version 2.14.1 (http://www.rproject.org) with R packages “Car”, “Coin”, “Corrgram”, “Gdata”, and “Psych”, “Pheatmap”. Statistical analysis of the numerical variables among the two groups was performed using Wilcoxon tests for nonparametric data. Principal component analysis (PCA) was performed to describe the distribution of all patients’ data. The volcano plot, including fold change and P value, showed the results of differential expression of lipid metabolites. The partial least squares-discriminant analysis (PLS-DA) was used to determine which lipid metabolites were a more reasonable explanation for the relationship among variables. PLS-DA was reconfirmed by the permutation testing that analyzed the separation distance for 1000 permutations. The PLS-DA or orthogonal partial least square-discriminate analysis (PLS-DA) model was cross-validated by reserved one-seventh of the variables, each sample being omitted once, to avoid overfitting. The variable importance in projection (VIP) value reflected the significance of those variables for the PLS-DA or OPLS-DA models. The VIP>1 was considered to be a statistically significant difference between the two groups. The distribution of components in the load matrix projection (S-plot) was carried out to describe the correlation (Y) using P value and the covariance (X). The heat map was presented by R software version 2.16.1 (R-tools technology Inc, 2015, USA) to visualize time-dependent changes of each lipid metabolite during the perioperative period. The analysis of variance (ANOVA) function of MetaboAnalyst (the Wishart Research Group, 2009, the University of Alberta) was performed to evaluate the number of lipid metabolites whose expression was significantly differential. The PLS-DA, heat map and volcano plot analysis were presented by MetaboAnalyst (http://www.metaboanalyst.ca). Heat map and volcano plot were performed by R sofware version 2.16.1 (http://www.rproject.org) with R packages “Pheatmap” and “ggplot2”.

## Results

### *Heat map relating to the relative level of lipid metabolites*

**Figure 1:**
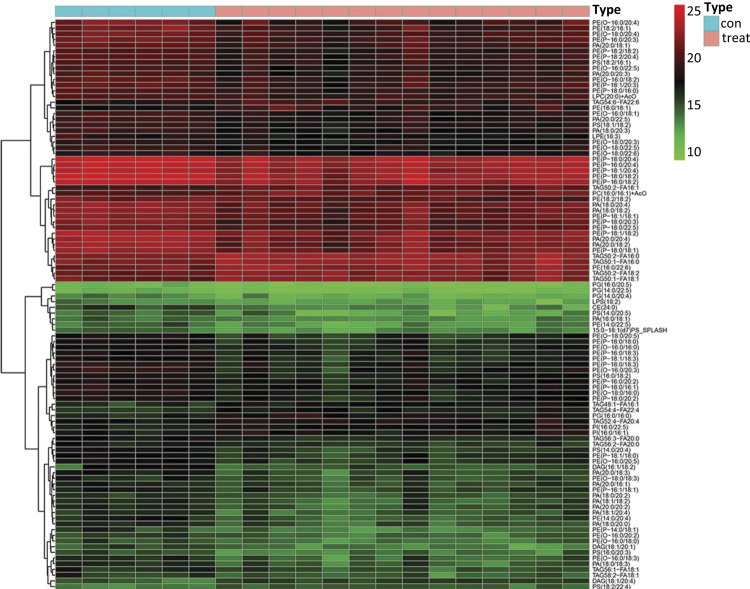
The heat map presented a top 100 of 1206 identified lipid metabolite. The hierarchical clustering analysis showed the healthy control group and pancreatic cancer group represented in the green and red color respectively.

A total of 1206 identified lipid metabolites that derived from two groups were visually analyzed by heat map using hierarchical clustering (Fig. 1). The results of hierarchical clustering analysis showed that the healthy control group and pancreatic cancer group (represented in the green and red color respectively) showed a sort of clustering in some areas. This phenomenon indicated that the distribution of lipid metabolites was different among the two groups. Furthermore, the heat map also presented variations in the relative concentrations of various lipid metabolites. This information provided an overview of lipid metabolites differences between the healthy control group and pancreatic cancer group; however, more detailed analysis was needed to better understand the lipidomics associated with pancreatic cancer.

### *Multivariate analysis of lipidomics data*

**Figure 2:**
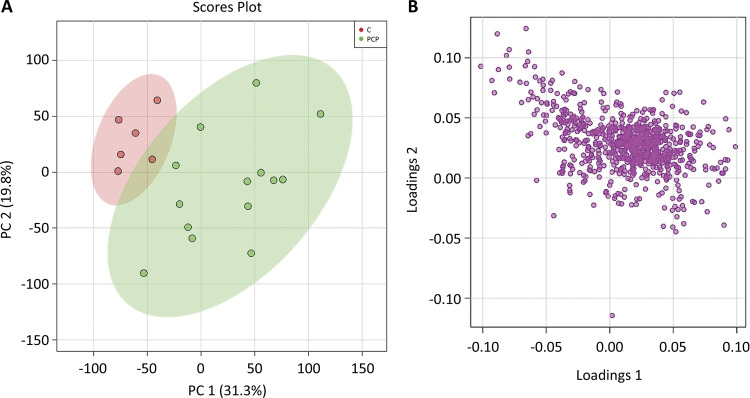
A) The principal components showed that the PC1 accounted for 31.3% of the total variance and the PC2 accounted for 19.8%. B) The abscissa of s-plot represents the correlation coefficient of principal component and metabolite, and the ordinate represents the correlation coefficient of principal component and metabolite. The purple dots represent metabolites.

The PCA model was constructed to find out the difference in lipid metabolites between the healthy control group and the pancreatic cancer group. In addition, the PCA model also illustrated the largest variation for related data that acquired from lipid metabolites, which used a few orthogonal latent variables. The PCA evaluated and determined the major sources of variance, and allowed samples to be clustered according to the similarity and difference of measurement parameters. Similarly, an overview of metabolomics data, including tendency and grouping, could also be described. The principal components, especially the first principal component (PC1) and the second principal component (PC2), were drawn in a score plot and any clustering tendency could be observed (Fig. 2). The PC1 accounted for 31.3% of the total variance that analyzed the sample data according to the plasm lipid level, while the PC2 accounted for 19.8%. And it may manifest that plasma lipid metabolites had a downward trend in patients with pancreatic cancer. The healthy control group and pancreatic cancer could be distinguished by the PCA score plot. The most valuable metabolites of PC1, including phospholipids and sphingolipids had significant difference between the two groups in the corresponding loading plot, and this characteristic could distinguish the healthy control group and patients with pancreatic cancer (Fig. 2).

### *Screening for potential tumor markers*

**Figure 3:**
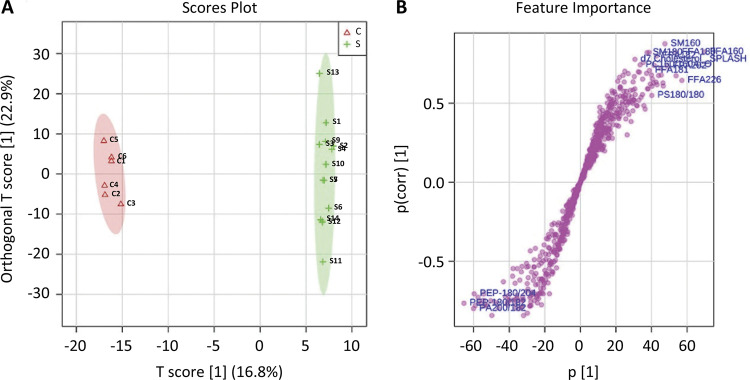
A) The PLS-DA derived score plot presented that component 1 accounted for 22.9% of the total variance and component 2 accounted for 16.8%. B) The abscissa of s-plot represents the correlation coefficient of principal component and metabolite, and the ordinate represents the correlation coefficient of principal component and metabolite. The purple dots represent metabolites.

In our study, the partial least-squares-based multivariate classification method and PLS-DA were performed to distinguish the healthy control group and pancreatic cancer group according to the identified lipid profiling datasets. The PLS-DA derived score plot presented that component 1 could clearly distinguish the two groups, which accounted for 22.9% of the total variance. In addition, component 2 accounted for 16.8% of the total variance (Fig. 3). All of these results implied that various changes in lipid metabolites were associated with pancreatic cancer. Hierarchical clustering analysis of the 30 lipids with the highest VIP scores showed that there are differences in lipid metabolites in patients with pancreatic cancer (Fig. 3).

### *Lipidomics analysis*

**Figure 4:**
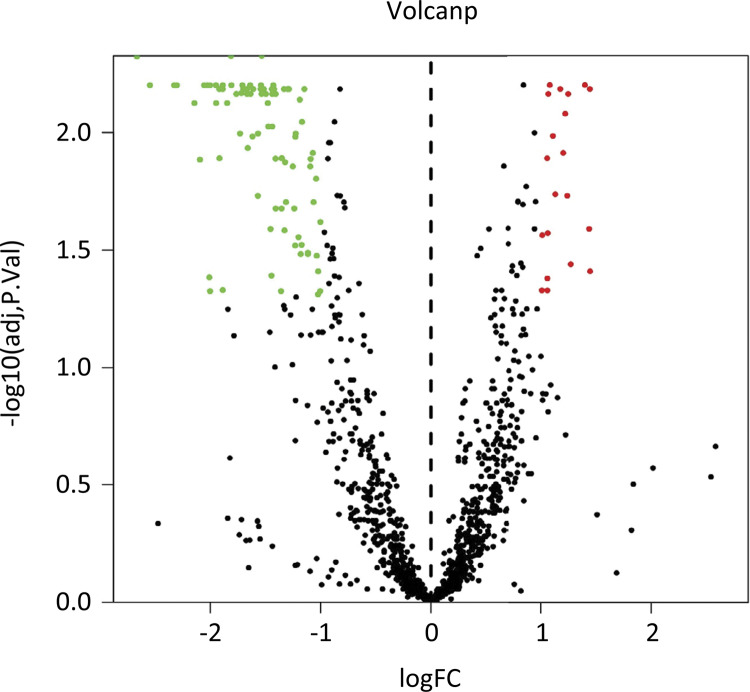
The volcano plot showed that the upregulated lipid metabolites contained 9 lipids and the downregulated lipid metabolites contained 79 lipids.

The volcano plot was used to visualize all variables with VIP>1 and presented the important variables with P<0.01 and |FC|>2 (red mark) (Fig. 4). The upregulated lipid metabolites contained diacylglycerol (DAG) (18:1/20:4), triacylglycerol (TAG) 50:2-fatty acid (FA) 16:0, TAG 50:2-FA 16:1, TAG 50:2-FA 18:2, TAG 48:1-FA 16:1, TAG 50:1-FA 18:1, phosphatidylinositol (PI) (16:0/22:5), phosphatidylserine (PS) (18:2/22:4) and phosphatidylcholine (PC) (16:0/16:1); however, the downregulated lipid metabolites contained 79 lipids (Table 1).

**Table 1:** The downregulated lipid metabolites contained 79 lipids (P <0.01, FC >2).

Lipid symbol	Results	Lipid symbol	Results	Lipid symbol	Results
CE(24:0)	C>S	PE(O-18:0/20:3)	C>S	TAG48:1-FA16:1	S>C
CE(22:0)	C>S	PE(O-18:0/20:4)	C>S	TAG50:1-FA18:1	S>C
TAG56:2-FA20:0	C>S	PE(O-18:0/20:5)	C>S	TAG50:2-FA16:0	S>C
TAG56:3-FA20:0	C>S	PE(O-18:0/22:5)	C>S	TAG50:2-FA16:1	S>C
DAG(16:1/18:2)	C>S	PE(P-14:0/18:1)	C>S	TAG50:2-FA18:2	S>C
DAG(18:1/20:1)	C>S	PE(P-16:0/18:2)	C>S	DAG(18:1/20:4)	S>C
PA(18:0/18:2)	C>S	PE(P-16:0/18:3)	C>S	PC(16:0/16:1)+AcO	S>C
PA(18:0/18:3)	C>S	PE(P-16:0/20:3)	C>S	PI(16:0/22:5)	S>C
PA(18:0/20:0)	C>S	PE(P-16:0/20:4)	C>S	PS(18:2/22:4)	S>C
PA(18:0/20:3)	C>S	PE(P-18:0/16:0)	C>S		
PA(18:0/20:4)	C>S	PE(P-18:0/16:1)	C>S		
PA(18:1/18:2)	C>S	PE(P-18:0/18:0)	C>S		
PA(18:1/20:4)	C>S	PE(P-18:0/18:1)	C>S		
PA(20:0/16:1)	C>S	PE(P-18:0/18:2)	C>S		
PA(20:0/18:1)	C>S	PE(P-18:0/18:3)	C>S		
PA(20:0/18:2)	C>S	PE(P-18:0/20:3)	C>S		
PA(20:0/18:3)	C>S	PE(P-18:0/20:4)	C>S		
PA(20:0/20:2)	C>S	PE(P-18:0/22:5)	C>S		
PA(20:0/20:3)	C>S	PE(P-18:1/18:0)	C>S		
PA(20:0/20:4)	C>S	PE(P-18:1/18:1)	C>S		
PA(20:0/22:5)	C>S	PE(P-18:1/18:2)	C>S		
PE(14:0/20:4)	C>S	PE(P-18:1/18:3)	C>S		
PE(14:0/22:5)	C>S	PE(P-18:1/20:3)	C>S		
PE(18:2/16:1)	C>S	PE(P-18:1/20:4)	C>S		
PE(O-16:0/16:0)	C>S	PE(P-18:2/18:2)	C>S		
PE(O-16:0/18:1)	C>S	PE(P-18:2/20:4)	C>S		
PE(O-16:0/18:2)	C>S	PG(14:0/20:4)	C>S		
PE(O-16:0/18:3)	C>S	PG(16:0/20:5)	C>S		
PE(O-16:0/20:2)	C>S	LPS(18:2)	C>S		
PE(O-16:0/20:3)	C>S	15:0-18:1(d7) PS_SPLASH	C>S		
PE(O-16:0/20:4)	C>S	PS(14:0/20:4)	C>S		
PE(O-16:0/20:5)	C>S	PS(14:0/20:5)	C>S		
PE(O-16:0/22:5)	C>S	PS(16:0/18:2)	C>S		
PE(O-18:0/16:0)	C>S	PS(18:1/18:2)	C>S		
PE(O-18:0/18:3)	C>S	PS(18:2/16:1)	C>S		

## Discussion

Pancreatic cancer is regarded as the most common and lethal disease of the digestive tract tumors. Altered metabolism and tumor microenvironment are considered as the Hallmarks of cancer^13^. Given the above considerations, deep thinking about metabolic dysregulation in patients with pancreatic cancer could be a novel discovery of tumor therapeutic targets^14^. It has been widely certified that detection of a metabolic profile in serum by mass spectrometry-based techniques was a feasible and sensitive tool to improve early diagnosis rate of malignant diseases, such as gastroenterological cancers^15^. Recently, metabolomics has been reported in many studies as an effective tool for the early diagnosis of pancreatic cancer^16-23^. In our study, we used liquid chromatography tandem mass spectrometry (LC-MS/MS) to analyze the difference of lipidomics for healthy control group and patients with pancreatic cancer, and then provided a novel method to describe the lipidomic characteristics of pancreatic cancer.

In addition, the plasma liposomes were regarded as a comprehensive assessment for patients with pancreatic cancer compared to healthy controls. Although our study did not show remarkable changes in the total number of lipid subtypes, the specific variation in individual molecular types also reflected selective remodeling of tumor progression. Specifically, as shown in figure 4, 4 FFAs were significantly increased in patients with pancreatic cancer compared with the healthy control group, including FFA (22:2), FFA (22:4), FFA (20:1) and FFA (20:2). FFAs played an irreplaceable role in numerous biological functions. FFAs were regarded as a source of energy and precursors of signal paths and cellular components. The function of different types of FFAs remained unclear on cell proliferation and apoptotic activity in patients with pancreatic cancer. Our study found that FFAs were significantly decreased in patients with pancreatic cancer. And thess results were consistent with previous research report by Zhang *et al*.^24^. The signaling pathways, relating to FAs, for the growth and death of pancreatic cancer cells have not been fully elucidated.

Our study showed that plasmalogen species alkyl-phosphatidy lethanolamine (PE-O) and phosphatidy lethanolamine plasmalogens (PE-P) had the highest VIP scores. According to previous research, PE levels were increased in breast cancer and pancreatic cancer cells^25^. In our study, we found that some of the PE-O and PE-P were significantly elevated. Therefore, we visualized the crucial parameters with P﹤0.01 and |FC|>2, most of the downregulated parameters were PE-O and PE-P.

PE linked to ethers differs from the more common PEs in the use of fatty alcohols instead of fatty acids^26^. Elevated levels of ether lipids have been widely reported in tumor tissues, and the discovery of important biological activities of unique ether-like lipids such as platelet activation factor (PAF) has greatly stimulated interest in these lipids^27,28^. Therefore, we found that partial ether-linked phospholipids were elevated in patients with pancreatic cancer, providing important new information for the specific types of ether-linked phospholipids involved in pancreatic cancer. The upregulation of ether lipids in tumor cells may be related to the proliferation of cancer cells and the increased tumorigenic potential^29^. Ether-related PE has been implicated in a series of developmental and tissue injury-related pathogenic processes, where they could regulate membrane fluidity and prevent oxidative stress by reducing the presence of acetal phospholipids in ROS, characterized by ethylene ether linkages in a vinyl ether linkage of the sn1 alky1 chain^30,31^.

In summary, we have identified a unique group of fatty acids associated with pancreatic cancer, providing new data for the study of lipid metabolomics in pancreatic cancer. Our results suggest that the damage of the residual lipolysis pathway of aldehyde fatty acid may be related to the development of pancreatic cancer. In addition, further studies are needed to determine the mechanism of these changes in aldehydes and to explore their potential clinical applications in pancreatic cancer. These studies will help better understand the aggressiveness of pancreatic cancer and identify therapeutic targets. However, this study also had some limitations, especially in the aspect of sample size. It was difficult to draw a definitive conclusion because of the small number of cases, and from our study we could only conclude that pancreatic cancer might be associated with lipidomics. Therefore, further research is needed to confirm the specific mechanism of lipomics associated with pancreatic cancer.

## Conclusion

There were lipid metabolomic differences in patients with pancreatic cancer, which could serve as potential tumor markers for pancreatic cancer.
