# Erratum: Differences in lipidomics may be potential biomarkers for early diagnosis of pancreatic cancer

**DOI:** 10.1590/s0102-865020200050000008err

**Published:** 2020-08-14

**Manuscript:** Differences in lipidomics may be potential biomarkers for early diagnosis of pancreatic cancer

**Publication:** Acta Cir Bras. 2020;35(5):e202000508

**Doi:** http://dx.doi.org/10.1590/s0102-865020200050000008

On the first page of the original publication, instead to this presentation by the authors:

Dehua Zhou^I^, Di Mu^II^, Ming Cheng^III^, Yuting Dou^IV^, Xianwei Zhang^V^, Zhensheng Feng^VI^, Guangting Qiu^VII^, Hua Yu^VII^, Yang Chen^VIII^, Hong Xu^VIII^*, Jian Sun^VIII^*, Ling Zhou^IX^*

Consider this:

Dehua Zhou^I^*, Di Mu^II^*, Ming Cheng^III^, Yuting Dou^IV^, Xianwei Zhang^V^, Zhensheng Feng^VI^, Guangting Qi^VII^, Hua Yu^VII^, Yang Chen^VIII^, Hong Xu^VIII^, Jian Sun^VIII^, Ling Zhou^IX^

